# DNA barcoding of Malaysian commercial snapper reveals an unrecognized species of the yellow-lined *Lutjanus* (Pisces:Lutjanidae)

**DOI:** 10.1371/journal.pone.0202945

**Published:** 2018-09-05

**Authors:** Adibah Abu Bakar, Eleanor A. S. Adamson, Lia Halim Juliana, Siti Azizah Nor Mohd, Chen Wei-Jen, Alias Man, Darlina Naim Md

**Affiliations:** 1 School of Biological Sciences, Universiti Sains Malaysia, Pulau Pinang, Malaysia; 2 Faculty of Science and Mathematics, Universiti Pendidikan Sultan Idris (UPSI), Tanjong Malim, Perak, Malaysia; 3 Institut Bioteknologi Marin, Universiti Malaysia Terengganu, Kuala Terengganu, Terengganu, Malaysia; 4 Institute of Oceanography, National Taiwan University, Taipei, Taiwan; 5 Fisheries Research Institute (FRI), Batu Maung, Pulau Pinang, Malaysia; Department of Agriculture and Water Resources, AUSTRALIA

## Abstract

Management of wild fisheries resources requires accurate knowledge on which species are being routinely exploited, but it can be hard to identify fishes to species level, especially in speciose fish groups where colour patterns vary with age. Snappers of the genus *Lutjanus* represent one such group, where fishes can be hard to identify and as a result fisheries statistics fail to capture species-level taxonomic information. This study employs traditional morphological and DNA barcoding approaches to identify adult and juvenile *Lutjanus* species harvested in Malaysian waters. Our results reveal a suite of species that differs markedly from those that have previously been considered important in the Malaysian wild-capture fishery and show that official fisheries statistics do not relate to exploitation at the species level. Furthermore, DNA barcoding uncovered two divergent groups of bigeye snapper (‘*Lutjanus lutjanus*’) distributed on either side of the Malay Peninsula, displaying a biogeographical pattern similar to distributions observed for many co-occurring reef-distributed fish groups. One of these bigeye snapper groups almost certainly represents an unrecognized species in need of taxonomic description. The study demonstrates the utility of DNA barcoding in uncovering overlooked diversity and for assessing species catch composition in a complicated but economically important taxonomic group.

## Introduction

The marine fisheries sector plays an important role in the Malaysian economy, contributing 1,483,000 metric tonnes of marine product which valued at RM 5.22 billion (USD 1.41 billion). This sector also supplies significant employment opportunities and foreign exports, and represents a source of protein for local rural populations. The marine waters surrounding the Malay coastlines that support this fishery are some of the most biodiverse regions in the world, located on the edge of the “coral triangle” and home to an estimated 1,400 marine fish species, including many endemics [1,2].

Approximately 200–300 species of marine fishes are landed in the major Malaysian landing sites, with an average of 50–100 species being displayed for sale daily in fish markets [3]. Additional species may appear seasonally, with certain species dominating market landings during the monsoons, while other permanent resident species of estuaries, bays and reef areas are landed throughout the year [3]. The diversity of wild species harvested and variety of fisheries operations in the country makes assembling accurate detailed catch data challenging. However, an important first step in understanding any fishery is to gather accurate information about which species are routinely targeted and landed.

Fish species identification is traditionally based on external morphological features including body shape, colour pattern, scale size and count, number and relative position of fins, fin rays and fin spines, or various relative measurements of body parts [4,5]. However, this approach sometimes requires solid ichthyological expertise to make positive identifications, and can be complicated when different life history stages (i.e. juvenile vs adult) have different appearances. Furthermore, due to low levels of morphological differentiation among some species, additional non-phenotypic information such as location or season of capture may be required for confident identification to species level.

Among the Family Lutjanidae, *Lutjanus* is by far the most speciose genus, with 71 species described to date [6,7] are widely distributed throughout inshore reef areas, sandy bays and estuaries. According to Chong et al. [2], there are 10 *Lutjanus* species present in Malaysian waters. Commonly known as snappers, fishes of the genus represent an important fishery resource in all the regions they occur [8–12]. Many species in the genus have highly similar morphologies, for example the red snappers [7,9,13] and the recently revised yellow-lined snapper complex [14]. Such high resemblance among species can make it difficult for fisheries officers and even for experienced taxonomists to reliably identify species based on external characteristics.

When traditional morphological characters prove problematic or inadequate in discriminating among species, molecular techniques such as DNA barcoding may aid in species recognition and identification [15]. This technique is based on the DNA sequence variation of a 650 base pair region of the mitochondrial cytochrome c oxidase subunit 1 (CO1) gene [16–18]. The primary goals of DNA barcoding are to assign unknown specimens to a species category and to enhance the discovery and description of new and cryptic species. In 2013, two new species of lutjanids from the Indo-West Pacific, *Lutjanus indicus* and *Lutjanus papuensis* were described by Allen et al. [19], with the help of information from DNA barcoding. More recent taxonomic work on the genus has also incorporated information on CO1 species barcodes, demonstrating the utility of barcoding for helping understand diversity in this genus [14].

Assessments made by Abu Talib et al. and WorldFish Center [20,21] indicate that the relative abundance of wild snappers has decreased sharply in Malaysia. However, a major limitation of harvest statistics in Malaysia, as in many other tropical multi-species fisheries, is the lack of proper identification of the catch at the species level. For example, the Department of Fisheries Malaysia (DoFM) uses the term red snapper (“Merah”) to collectively refer to two species, *Lutjanus malabaricus* and *Lutjanus sebae*, while “remong/kunyit-kunyit” is applied to species in the yellow-lined group including *Lutjanus lutjanus* and *Lutjanus vitta*. Species within these groups may well have different vulnerabilities to fishing activities, and therefore could require different management policies to ensure sustainable harvest. It is impossible to develop conservation plans and long-term management strategies without knowing what species are involved, and preferably also whether subpopulations exist.

In this study we collected *Lutjanus* specimens from landing sites across Malaysia and identified them to species level using a combination of morphological and DNA barcoding information. We quantify the number of species commonly harvested at commercial landing sites, examine the reliability of morphological characters for species level identification, and detail the presence of an unrecognized species of *Lutjanus* among the Malaysian snapper catch.

## Method

### Ethics statement

All marine life examined in this study were already dead upon inspection. Permission to undertake surveys in Malaysia was granted by the Fisheries Research Institute, Batu Maung, Penang as part of a collaborative project with Universiti Sains Malaysia.

### Specimen collection

Specimen collection aimed to obtain representative samples of all *Lutjanus* species commonly occurring in commercial Malaysian catches. DoFM data indicates there is significant regional variation in species composition of lutjanid catch [3], therefore specimen collection was undertaken as widely as possible around the coast of Malaysia. In total, 260 fishes were collected from 25 landing sites during the period 2012–2014, representing catches harvested from the Strait of Malacca, South China Sea, Sulu Sea and Celebes Sea (Table 1). Each landing site was visited on two separate occasions and all *Lutjanus* specimens that were encountered were sampled. Specimens were first identified based on morphological criteria (color, meristic traits) and classified as best as possible to species level [8,14]. Photographs of each fresh specimen were taken for inventory purposes before fin clips were sampled (from all 260 samples collected) and stored in 98% ethanol prior to molecular work. Full specimens were then fixed in 10% formalin for 5 days before transfer to 70% alcohol for long-term storage and were deposited at the Centre of Marine and Coastal Studies (CEMACS) Collection Centre, Batu Maung and Museum Biodiversity, Universiti Sains Malaysia, Pulau Pinang. In some cases, *L*. *malabaricus* and *L*. *johnii* specimens were too large to preserve, therefore only photographs and fin clips were retained.

**Table 1:** Locations of the twenty-five landing sites visited in this study, including the adjacent coastline (WP = West Peninsular, EP = East Peninsular, B = Borneo) and the marine region where the fish catch originated (M = Strait of Malacca, S = Strait of Johor, WSC = west South China Sea, ESC = east South China Sea, SS = Sulu Sea, CS = Celebes Sea). Numbers in first column and species abbreviations refer to Fig 1.

No.	Locality	Specimens collected	Latitude	Longitude	Coastline	Marine Region	Species collected
1.	Kuala Perlis, Perlis	10	6°24'01"N	100°7'49"E	WP	M	LJ, LL2, LLM, LQ, LV, LX
2.	Kuala Kedah, Kedah	7	6°08'00"N	100°18'0"E	WP	M	LJ, LL2, LM,
3.	Batu Maung, Penang	6	5°18'08.1"N	100°17'16.9"E	WP	M	LI, LM
4.	Kuala Sepetang, Perak	6	4°49'15.8"N	100°42'25.5"E	WP	M	LA, LJ, LM
5.	Lumut, Perak	6	4°15'54.4"N	100°39'57.1"E	WP	M	LE, LJ
6.	Bagan Panchor, Perak	10	4°31’42.63”N	100°38’21.64”E	WP	M	LI, LJ, LL2, LV
7.	Kuala Gula, Perak	10	4°56'15.8"N	100°28'06.5"E	WP	M	LJ
8.	Sg. Besar, Selangor	10	3°39’50.10”N	100°59’14.35”E	WP	M	LJ
9.	Kuala Selangor, Selangor	6	3°21’0.05” N	101°15’10.93”E	WP	M	LA, LM
10.	Pasir Panjang, Negeri Sembilan	10	2°24'58.1"N	101°56'33.8"E	WP	M	LJ
11.	Kuala Sg. Baru, Melaka	6	2°21'34.6"N	102°02'23.5"E	WP	M	LJ, LR
12.	Sg. Muar, Johor	10	2°03'10.3"N	102°34'17.5"E	WP	M	LJ
13.	Kongkong, Johor	10	1°31'8.01"N	103°59'52.16"E	WP	S	LA
14.	Mersing, Johor	12	2°26'01.1"N	103°50'10.6"E	EP	WSC	LJ, LE, LM, LSB
15.	Chendering, Terengganu	10	5°15’51.54”N	103°11’8.24”E	EP	WSC	LJ
16.	Marang, Terengganu	10	5°12’24.83”N	103°12’27.00”E	EP	WSC	LM
17.	Pulau Kambing, Terengganu	12	5°19’21.92”N	103°746.37”E	EP	WSC	LM, LQ, LR, LSB
18.	Kampung Rhu 10, Terengganu	6	5°35’0.04”N	102°50’16.54”E	EP	WSC	LJ, LR
19.	Tok Bali, Kelantan	6	5°52'37.3"N	102°27'25.2"E	EP	WSC	LL1, LM
20.	Kuala Besar, Kelantan	6	6°12'22.1"N	102°14'04.0"E	EP	WSC	LJ, LM
21.	Kota Kinabalu, Sabah	60	5°58’59.12”N	116°4’18.32”E	B	ESC	LA, LB, LC, LD, LF, LJ, LR, LV
22.	Kudat, Sabah	6	6°52’37.69’N	116°50’57.48”E	B	SS	LA, LB, LE LM
23.	Sandakan, Sabah	5	5°51’14.07”N	118°7’46.51”E	B	SS	LJ
24.	Tawau, Sabah	10	4°14’ 32.63”N	117°53’2.04”E	B	CS	LJ
25.	Mukah, Sarawak	10	2°53'52.5"N	112°05'44.7"E	B	ESC	LQ
		Total = 260					

LA-*L*.*argentimaculatus* LL2-*L*.*lutjanus 2*

LB-*L*.*bohar*     LLM-*L*.*lemniscatus*

LC-*L*.*carponotatus*  LM-*L*.*malabaricus*

LD-*L*.*decussatus*   LQ-*L*.*quinquelineatus*

LE-*L*.*erythropterus*  LR-*L*.*russelli*

LF-*L*.*fulviflamma*   LSB-*L*.*sebae*

LI-*L*.*indicus*    LV-*L*.*vitta*

LJ-*L*.*johnii*     LX-*L*.*xanthopinnis*

LL1-*L*.*lutjanus 1*

### DNA extraction, amplification and sequencing

DNA was extracted following the CTAB extraction method [22]. PCR amplification of the CO1 locus was performed using combinations of primers F1, F2, R1 and R2 [23] in 25.0 μl reaction volumes including 8.37μl molecular grade water, 1.25μl 10X PCR buffer, 1.0μl of MgCl_2_ (25mM), 0.25μl of each primer (10mM), 0.5μl of Intron *i*-Taq^™^ plus DNA Polymerase (5U/μl) and 0.75μl of template DNA. The PCR conditions consisted of 95°C (2 min), 35 cycles of 94°C (45 sec), annealing temperature 47.9–48.0°C (45 sec), extension step at 72°C (45 sec) followed by a final extension at 72°C (2 min). PCR products were visualized in a 2% agarose gel and subsequently sent to sequencing service provider NHK Bioscience Solution Sdn. Bhd.

### Data analysis

Sequences were trimmed and aligned using MEGA ver. 6.06 software [24]. The quality and length of CO1 sequences and the presence of stop codons or indels in the reading frame were checked to ensure the data set did not contain errors or pseudogenes. Confirmation of species identifications based on morphology was conducted by comparing CO1 barcode sequences to the Genbank database [25] and BOLD system database [26] with reference to recently published taxonomic works [14]. Similarity thresholds of 99% were used to assign specimens to species level. Pairwise genetic distances within and among species were calculated under the Kimura 2-parameter (K2P) model [27] performed in MEGA software. The same software was used to cluster CO1 haplotypes into a Neighbour-Joining (NJ) phylogeny, employing 1000 bootstrap replicates. A member of the genus *Pristipomoides* was included to represent genetic divergence at a higher taxonomic level (among genera within the Lutjaninae subfamily). In one case, where divergent lineages of the yellow-lined snapper complex [14] could not be classified to species level, a further NJ tree was constructed to illustrate diversity in the group using available sequence data for members of the complex [14].

## Results

We recovered 17 species among the 260 *Lutjanus* specimens sampled across the 25 landing sites (Table 1), including a divergent and unrecognised group of *L*. *lujanus*. One hundred and seventy-nine specimens were positively identified using morphology, while the remaining 81 which consisted of red snapper juvenile specimens, were identified using a combination of morphological and genetic information. Among the total samples, a *L*. *xanthopinnis* specimen was only observed once, while *L*. *johnii* was the most commonly occurring lutjanid species with 100 individuals collected across all sampling localities. The highest number of species was collected at Kota Kinabalu, Sabah (eight species); in contrast only a single species was encountered at 10 different landing sites (refer Table 1). Importantly, our survey failed to encounter *L*. *monostigma*, a species that is listed as commercially important in Malaysia [28].

**Fig 1:**
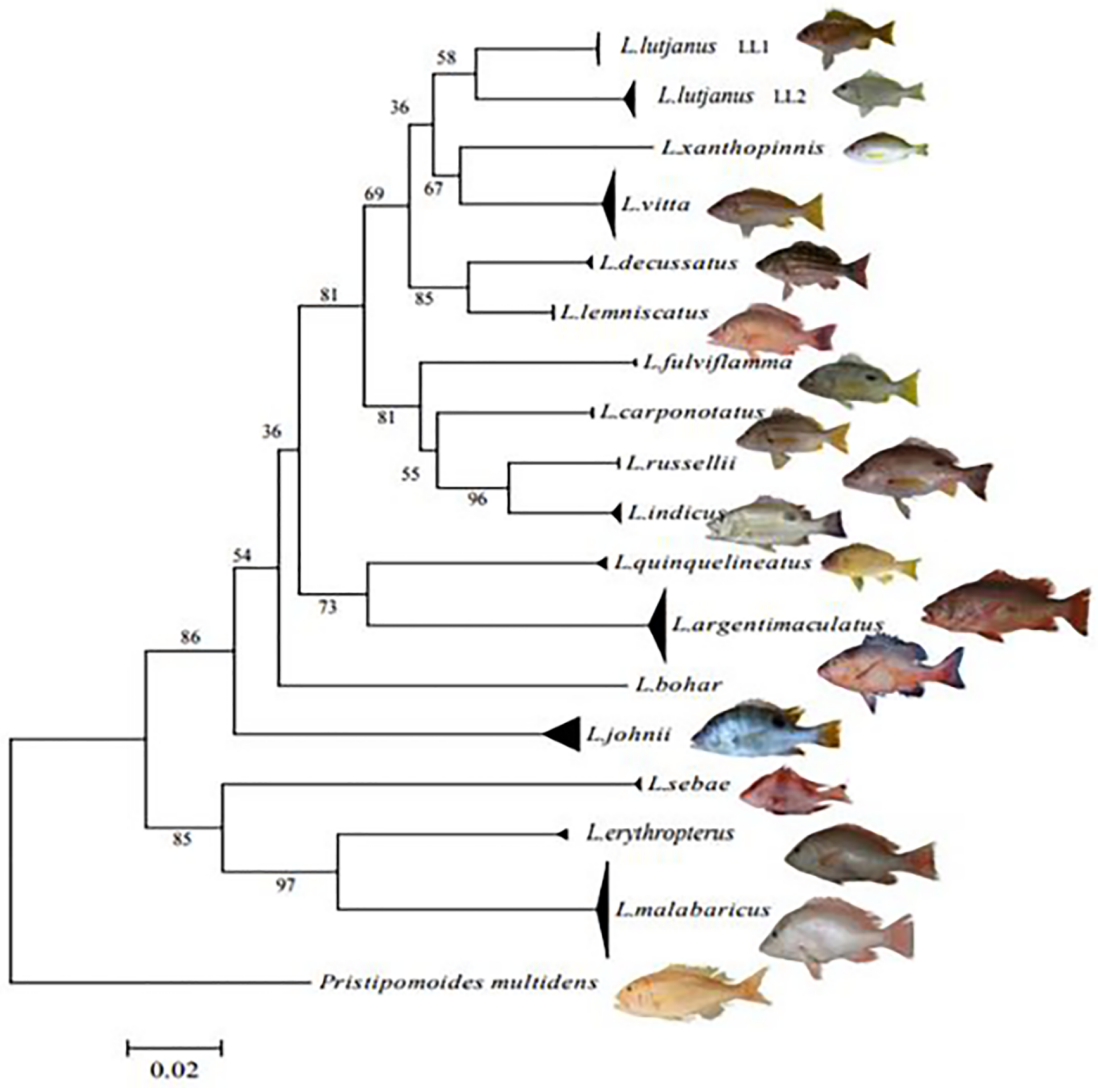
Neighbour-Joining (NJ) tree of CO1 barcodes for all *Lutjanus* species collected at Malaysian landing sites. Tips representing sequences for all individuals sampled have been collapsed into species clades to simplify the illustration of diversity within and among species, except in the case of *L*. *Lutjanus* (*LL1* & *LL2*) where two groups were retained to show the presence of two divergent lineages, see Fig 2. All bootstrap values for conspecific groups were > 50%, the scale bar indicates percent divergence calculated under the K2P model.

All samples were successfully sequenced for the CO1 region, and no evidence was observed in the final 651 base pair alignment to indicate the presence of pseudogenes in the data set. The alignment had 213 variable sites (33%), with 196 (30%) parsimoniously informative sites. Representative haplotypes of each species were submitted to GenBank (MG002612-MG002629). Within the 17 species, average intraspecific K2P divergence ranged between 0 to 0.4% while interspecific divergence ranged from 4.5% to 21.3% (Table 2). Most nodes in the NJ tree had bootstrap support of >50% (Fig 1). Ten specimens that were morphologically identified as *L*. *malabaricus* were identified as *L*.*erythropterus* in GenBank. As GenBank does not have other verification information such as picture of specimen and these two species also have been reported to be always mislabeled [29], work is currently underway in our lab to resolve this issue.

**Table 2:** Average K2P divergences between CO1 barcodes of the 17 *Lutjanus* species sampled from Malaysian landing sites. Intraspecific comparisons are indicated by shaded text. Minimum and maximum interspecific divergence values are bolded, highlighting comparatively low divergence between *L*. *decussatus* and *L*. *lemniscatus* and high divergence between *L*. *malabaricus* and *L*. *vitta*. For full species names see Fig 1.

	*LA*	*LB*	*LC*	*LD*	*LE*	*LF*	*LI*	*LJ*	*LL1*	*LL2*	*LLM*	*LM*	*LQ*	*LR*	*LS*	*LV*	*LX*
*LA*	0.004																
*LB*	0.174	0.000															
*LC*	0.141	0.141	0.000														
*LD*	0.131	0.147	0.094	0.003													
*LE*	0.175	0.167	0.179	0.170	0.004												
*LF*	0.148	0.158	0.077	0.083	0.189	0.000											
*LI*	0.161	0.146	0.068	0.093	0.182	0.089	0.002										
*LJ*	0.149	0.165	0.154	0.133	0.169	0.162	0.161	0.006									
*LL1*	0.140	0.144	0.107	0.090	0.182	0.116	0.115	0.139	0.001								
*LL2*	0.131	0.125	0.093	0.083	0.184	0.105	0.105	0.132	0.061	0.004							
*LLM*	0.138	0.153	0.098	**0.045**	0.181	0.099	0.107	0.154	0.082	0.086	0.000						
*LM*	0.181	0.174	0.188	0.179	0.107	0.169	0.187	0.192	0.193	0.191	0.182	0.003					
*LQ*	0.115	0.135	0.134	0.141	0.175	0.145	0.142	0.158	0.122	0.127	0.150	0.195	0.003				
*LR*	0.161	0.150	0.078	0.090	0.171	0.101	0.047	0.159	0.117	0.097	0.095	0.179	0.138	0.000			
*LS*	0.195	0.197	0.179	0.191	0.171	0.179	0.177	0.188	0.211	0.210	0.190	0.165	0.190	0.179	0.001		
*LV*	0.151	0.150	0.105	0.086	0.200	0.114	0.109	0.163	0.075	0.085	0.074	**0.213**	0.131	0.109	0.198	0.004	
*LX*	0.134	0.149	0.111	0.089	0.185	0.117	0.117	0.147	0.084	0.094	0.086	0.193	0.145	0.120	0.205	0.076	n/a

### Yellow-lined snapper complex

During the collection and morphological identifications a total of 34 specimens from four sites were tentatively identified as *Lutjanus lutjanus*, a species in the yellow-lined snapper complex [14]. DNA barcoding of these specimens subsequently uncovered two genetically distinct and well supported monophyletic lineages (58% bootstrap support) with deep genetic divergence (K2P = 6.1%) between them (Table 2, Fig 1). This divergence is an order of magnitude higher than any other intraspecific value observed in this study (see Table 2), and we suggest that the two lineages likely represent different species within the yellow-lined complex but additional investigation is required. The lineages were not sympatric. One group (*LL1*) corresponded to 15 individuals collected from the South China Sea fishery (site 19) on the eastern coastline of Peninsular Malaysia, while the second group, composed of 19 individuals (*LL2*), was caught in the Strait of Malacca (sites 1, 2 & 6) on the western coast of the Peninsula.

**Fig 2:**
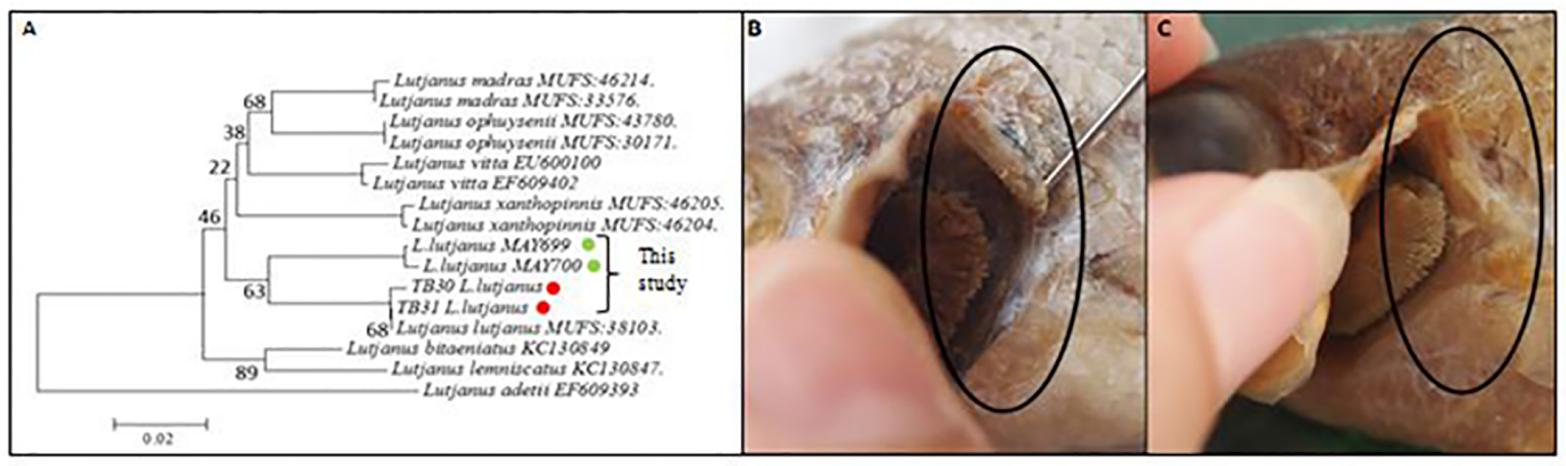
**A**: Neighbour-Joining (NJ) tree of CO1 barcode data for all species of the yellow-lined snapper complex excluding *L*. *mizenkoi* where no sequence data was available. **B & C**: Left ventral view of the cleithrum region for each of the two *L*. *lutjanus* phenotypes that were observed, showing difference in articulation between the supracleithrum and the cleithrum; **B** in *LL2* specimens from site 1 the elements are somewhat unattached, while in *LL1* and *LL2* specimens from sites 2, 6, 19 (**C**) the elements are fully fused.

In order to describe our *L*. *lutjanus* diversity in the context of the yellow-lined snapper complex including the newly described *L*. *xanthopinnis* [14], we gathered detailed morphological data (S1 Appendix) and compared CO1 sequences with other members of this group (Fig 2, Table 3). Both groups had morphological features and meristic counts that were diagnostic of *L*. *lutjanus*, confirming that neither lineage corresponds to other recognised species within the complex. Close examination of our two groups revealed that they differed in body depth to total length ratio (2.00–3.50 for *LL1*, compared to 3.10–3.78 for *LL2*). Further examination revealed that *LL2* specimens from site 1 also exhibited a noteworthy difference in supracleithrum-cleithrum articulation when compared to both *LL1* and *LL2* individuals from all other locations (see Fig 2B & 2C). While this characteristic did not correspond with monophyletic groups based on genetic information, it does provide further evidence that populations of *L*. *lutjanus* are not uniform across the collection area. Among the yellow-lined complex, our two *L*. *lutjanus* groups appear to be sister taxa (Fig 2A), with South China Sea specimens (*LL1*) corresponding to the species previously barcoded as *L*. *lutjanus* (MUFS: 38103, Japan,) [14]. We have also observed comparable levels of genetic divergence between the two groups at nuclear loci (unpublished data), further supporting the taxonomic separation of these two groups.

**Table 3:** Average K2P divergences between CO1 barcodes of the yellow-lined lutjanids, including the two bigeye snapper *Lutjanus lutjanus* lineages (*LL1* & *LL2*, red and green dots respectively) encountered in this study.

	*L*. *adetii*	*L*. *bitaeniatus*	*L*. *lemniscatus*	*LL1*	*LL2*	*L*. *madras*	*L*. *ophuysenii*	*L*. *vitta*	*L*. *xanthopinnis*
*L*. *adetii*	n/a								
*L*. *bitaeniatus*	0.141	n/a							
*L*. *lemniscatus*	0.167	0.043	0.002						
*LL1*	0.173	0.077	0.085	0.002					
*LL2*	0.154	0.069	0.082	0.059	0.003				
*L*. *madras*	0.163	0.073	0.078	0.063	0.073	0.003			
*L*. *ophuysenii*	0.157	0.071	0.076	0.063	0.081	0.049	0.000		
*L*. *vitta*	0.160	0.069	0.072	0.072	0.077	0.056	0.060	0.007	
*L*. *xanthopinnis*	0.159	0.079	0.086	0.074	0.088	0.070	0.072	0.068	0.003

## Discussion

### Lutjanus fishery in Malaysia

FishBase [28] suggests that there are 26 *Lutjanus* species present in Malaysian waters, of which eight are listed as native and of commercial value (*Lutjanus argentimaculatus*, *L*.*decussatus*, *L*.*fulviflamma*, *L*.*lutjanus*, *L*.*monostigma*, *L*.*quenquelineatus*, *L*.*russelli* and *L*.*vitta*). We encountered seven of these, but failed to encounter the eighth, *L*. *monostigma*. Lutjanids are non-migratory, and the limited catch data available from the DoFM suggests there is no seasonal variation in *Lutjanus* catches [3], so we consider our failure to recover *L*. *monostigma* is unlikely to be influenced by seasonality of sampling. Rather, our results reveal that there is still much to be quantified about the composition of the *Lutjanus* fishery in Malaysian coastline. In total, we encountered 17 species, more than twice the number of ‘commercial species’ listed on FishBase and routinely documented by the DoFM in their catch statistics, demonstrating that many more *Lutjanus* species than expected are subject to commercial fishing pressure in Malaysia.

The composition of species varied greatly in catches from different areas around the coastlines of Malaysia and from different marine regions. The most commonly encountered species, *L*. *johnii*, was encountered broadly in all areas, as was *L*. *malabaricus*. A number of species were confined to the Strait of Malacca (*L*. *indicus*, *L*. *xanthopinnis*, *L*. *lutjanus-LL2*), the west South China Sea (*L*. *sebae*, *L*. *lutjanus-LL1*) or the east South China Sea/Borneo fisheries (*L*. *bohar*, *L*. *carponotatus*, *L*. *decussatus*). The differences in catches may reflect different habitats and corresponding species communities around the Malaysian coastline, but may also be influenced by fishing operations targeting different species in response to market pricing and demand; residents of East Malaysia in Sabah and Sarawak prefer and can afford large fishes (seafood is commonly cheaper there), while Peninsula residents prefer to purchase smaller individuals, hence supply is biased in favour of smaller species.

Recent annual fishing statistics [3] indicate lutjanids contribute 5.3% (15,013 tonnes) of the overall marine fish catch (282,760 tonnes). The DoFM pools *Lutjanus* catch data into four local name categories: “Jenahak” (*L*. *johnii*), “Merah” (*L*. *malabaricus + L*. *sebae*), “Tanda” (*L*. *russelli*) and “Remong/Kunyit-kunyit” (*L*. *lutjanus + L vitta*), with the “Merah” group officially accounting for over half the total *Lutjanus* catch in 2013. Our landing site sampling revealed a different picture of catch composition (refer S2 Appendix), with almost 38% of all individuals encountered identified as *L*. *johnii* (“Jenahak”). We also encountered sub-adult *L*. *johnii* for sale as “Tanda”, suggesting misclassification of *L*. *johnii* is likely explains the discrepancy between low official catch statistics and the relatively high number of this species actually available for sale.

The categories used by the DoFM directly reference prominent body colour patterns. “Jenahak” usually possess silvery or gold coloured bodies; “Merah” are red; “Tanda” refers to individuals that show a prominent ventral spot found above the lateral line below the anterior dorsal-fin rays while the “Remong/Kunyit-kunyit” group contains snappers of yellowish colouration. These classifications are complicated because they rely on common colour characteristics of lutjanids that don’t correspond consistently with individual species or groups of species. We discovered that the “Tanda” group (ventral spot) contained sub-adult *L*. *johnii* (“Jenahak” group). Local fishermen could not differentiate between *L*. *russelii* ('Tanda" fishes) and *L*. *johnii* (“Jenahak” fishes) because both species’ juveniles show ventral spot. We also observed cases where adult *L*. *russellii* (“Tanda”) were included in the “Merah” category (adult *L*. *russellii* lose their spot and are red).

The misuse of common names with respect to species designation clearly indicates that catch statistics do not reflect the numbers of individual species harvested. This means that the economic importance of some species is likely to have been severely underestimated, especially in the case of *L*. *johnii* which was the most common species encountered here, despite having low catch statistics and not being listed as a commercial species in Malaysia on Fishbase [28].

Historical data from the DoF indicates no significant decline in overall fish capture,. However, in the case of lutjanids, as these assessments have been made based on pooled catch data they offer no real insight into changes over time for individual species. It is quite possible that in the absence of species-specific records and amid the confusion of accurate species identifications, over estimation of stock units and species abundances for some species may also have occurred. Other sources have noted specific declines from 2003 to 2006 in lutjanid catches [20,21] and we can be certain based on our survey that many more species are being exploited by Malaysian fishing operations than have previously been documented.

### Application of DNA barcoding

Here we demonstrate how DNA barcoding can be applied effectively as a tool to identify *Lutjanus* species from mixed catches. Our results identify *Lutjanus* species present in the Malaysian catch that were not formerly known to be targeted by fisheries activities, uncover a new lineage of one economically important group that is likely to represent an unrecognized species, and highlight the inadequacy of current fisheries statistics for capturing species level information.

These findings are not surprising given the diversity of lutjanid fishes and the nature of information currently available on the lutjanid fishery. The genus *Lutjanus* is large, and it’s clear from the recent addition of newly described species and from our own results that taxonomy within the group is not yet resolved. Identification to species-level using traditional morphological characters can be difficult for members of the genus, especially in cases where juvenile and adult specimens exhibit variation in coloration, and this challenge has resulted in catch statistics that are not reflective of species level composition.

DNA barcoding as demonstrated here provides a relatively easy means for identifying fishes in the group [16,23,29], avoiding difficulties associated with field morphological identification. The approach offers great potential as a tool for high-quality fisheries monitoring, and regular application would yield high quality information about the ongoing composition and exploitation for *Lutjanus* species in Malaysian waters. The discovery here of the likely presence of two ‘*Lutjanus lutjanus*’ species will require comprehensive taxonomic work to determine the true species status of both lineages. Nevertheless, without DNA barcoding, the presence of two groups may never have been uncovered, demonstrating the power of the barcoding approach to reveal unrecognized diversity.

### Unrecognized taxonomic diversity in the yellow-lined snapper complex

The *Lutjanus* yellow-lined complex received recent attention in Iwatsuki et al. [14], including the description of a new species, *L*. *xanthopinnis*. Results of our study indicate the likely presence of yet another species in the group. The two lineages uncovered here (*LL1* & *LL2*) all had meristic characters diagnostic of *L*. *lutjanus* as opposed to other members of the complex (for example 4–5 scale rows above lateral line). However, there was comparatively large genetic distance (6.1%) and general morphological differences (for example body depth) between the two, strongly suggesting at least one group is likely to represent an unrecognised species.

Although we were unable to determine from morphology which of our two *L*. *lutjanus* lineages may constitute a new species of *L*. *lutjanus*, DNA barcodes for our *LL1* South China Sea specimens were very similar (0.03%) to *L*. *lutjanus* barcodes from Japan. *Lutjanus lutjanus* Bloch 1790 was probably originally described from an Indonesian specimen [6], and while it is currently recognized as occurring over a large area in the Indian and Pacific Oceans, there have been many synonymies for descriptions dealing with both Indian and Indo-Pacific taxa. Without examining original material we cannot be sure whether the lineages we uncovered may correspond with previously described taxa, or whether at least one may constitute a species new to science. We can however be confident that at present at least one lineage represents a currently unrecognized taxon.

The geographical distribution of the two groups is interesting, especially as they appear to be sister taxa (Fig 2A). The *LL2* group occurred only to the west of Peninsular Malaysia in the Strait of Malacca, while the *LL1* group occurred to the east in the South China Sea. It is likely that the divergence between the two *L*. *lutjanus* taxa observed here is the result of the same processes that have shaped the biogeography of other reef fishes in the region, processes that may involve differences in ocean temperature and sedimentation, open-ocean distances between reefs, direction of ocean currents, or long periods of limited connectivity during times of higher sea level [30–34]. Indeed, such factors are demonstrated to have influenced historical partitioning of marine organism within the Indo-Pacific [35–40]. As well as being relevant to ecological and evolutionary studies, this biogeographical structuring has important general implications for Malaysian fisheries, as it is likely to heavily influence the composition of fish communities and hence species catches in different marine regions off the Malaysian coastlines.

## Conclusion

This study demonstrates how DNA barcoding can be employed to gain new knowledge of a multi-species capture fishery, revealing the likely presence of an unrecognised species and the undocumented exploitation others. We hope that the diversity documented here will provide a useful resource for future researchers and managers seeking accurate information on the species composition of the *Lutjanus* fisheries, and ultimately aid the formulation of effective management plans. For countries such as Malaysia with diverse and abundant marine fauna, accurate species identification will be key to unravelling and conserving the wealth of hidden biological diversity.

## Supporting information

S1 Appendix Morphological data for LL1 and LL2. (DOC)

S2 Appendix Species composition of specimens obtained. (DOC)
