# Supplementary material for: DNA barcoding of Malaysian commercial snapper reveals an unrecognized species of the yellow-lined Lutjanus (Pisces:Lutjanidae)
Source: PLoS One. 2018 Sep 5;13(9):e0202945. doi: 10.1371/journal.pone.0202945 (PMC6124743; doi:10.1371/journal.pone.0202945)
Supplement: S1 Appendix — (DOC) [file pone.0202945.s001.doc]

**S1 Appendix**

**Morphological data for LL1 and LL2**

S1 Table (a): Proportional measurements (as percentage of standard length -SL with range [mean]) for

*Lutjanus lutjanus 1* (LL1)*, L. lutjanus* (LL2).

| **No** |  | **LL1**  **(n=9)** | **LL2**  **(n=9)** |
| --- | --- | --- | --- |
| 1 | Body depth (BD) | 32-42 (38) | 28-33 (31) |
| 2 | Body depth at 1st anal spine (BD1) | 29-36 (32) | 26-29 (28) |
| 3 | Head length (HL) | 30-38 (33) | 30-35 (33) |
| 4 | Snout length (SL) | 8-11 (10) | 9-11 (10) |
| 5 | Orbit diameter (OD) | 8-11 (10) | 9-12 (10) |
| 6 | Interorbital width (IOW) | 6-9 (8) | 8-10 (9) |
| 7 | Interobital width + membrane (IOWm) | 8-10 (9) | 9-11 (10) |
| 8 | Upper jaw length (UJL) | 15-19 (16) | 6-16 (14) |
| 9 | Suborbital depth (SOD) | 3-6 (5) | 5-6 (5) |
| 10 | Caudal peduncle depth (CPD) | 10-14 (12) | 10-12 (11) |
| 11 | Caudal peduncle length (CPL) | 14-20 (17) | 15-21 (18) |
| 12 | Predorsal length (PDL) | 32-42 (37) | 35-42 (39) |
| 13 | Preanal length (PAL) | 73-88 (78) | 71-81 (75) |
| 14 | Prepelvic length (PPvL) | 33-43 (37) | 35-43 (38) |
| 15 | Dorsal-fin base (DFB) | 46-62 (55) | 45-56 (52) |
| 16 | Anal-fin base (AFB) | 13-19 (15) | 12-15 (14) |
| 17 | Caudal-fin length (CFL) | 19-28 (23) | 20-28 (24) |
| 18 | Pelvic-fin spine length (PvSL) | 13-30 (16) | 11-14 (12) |
| 19 | Pelvic-fin length (PvL) | 19-23 (22) | 18-23 (21) |
| 20 | Pectoral-fin length (PFL) | 19-30 (25) | 19-30 (27) |

S1 Table (b): Meristic counts for *Lutjanus lutjanus 1* (LL1) and  *L. lutjanus 2* (LL2).

| No |  | **LL1** | **LL2** |
| --- | --- | --- | --- |
| 1 | Dorsal-fin rays (DFR) | XII, 12 | XII, 12 |
| 2 | Anal-fin rays (AFR) | III, 8 | III, 8 |
| 3 | Pectoral-fin rays (PFR) | 16-17 | 16-17 |
| 4 | Lateral-line scales (LC) | 28-35 | 28-35 |
| 5 | Horizontal scale rows above lateral line (SAL) | 4-5 | 4-5 |
| 6 | Scale rows on cheek (SC) | 5-8 | 5-8 |
| 7 | Scale rows above / below lateral line (SAL/SBL) | 4-5/8-9 | 4-5/8-9 |
| 8 | Gill rakers, upper and lower  limb totals (rudiments)= total including rudiments (GR) | 10-11(1-2) +  10-11 (1-2)=  20-22 | 9-10(2-3) +  8-9(2/3)=  17-19 |

**S1 Figure**


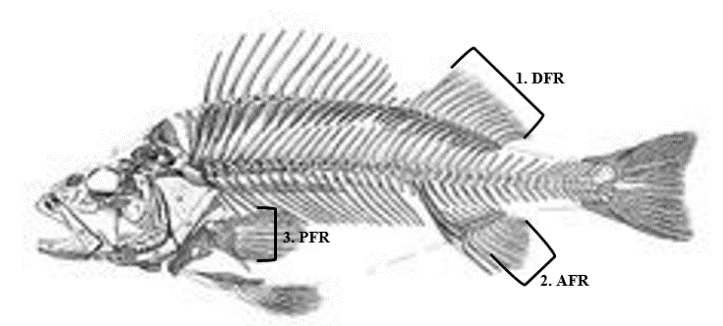

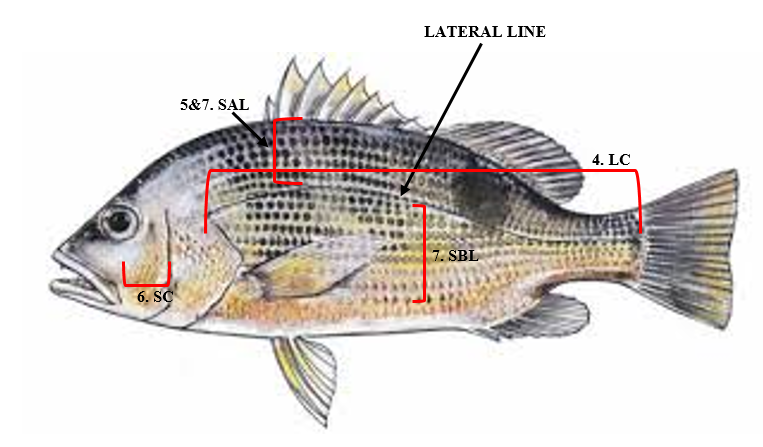

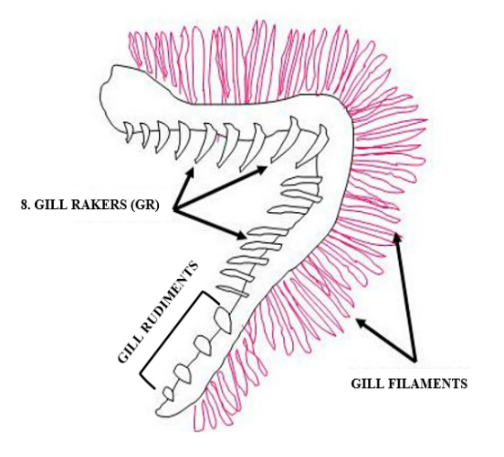

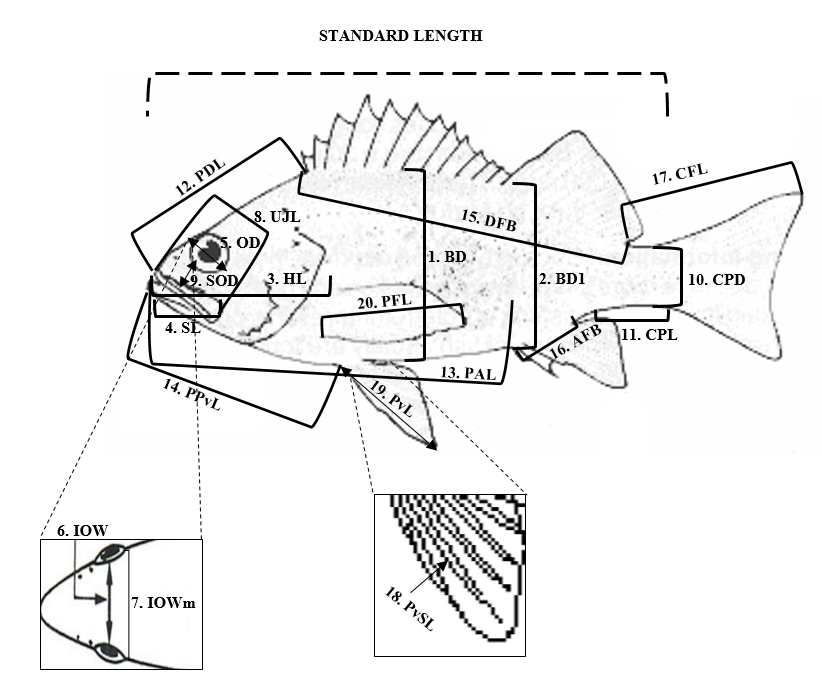


**(SL)**
