# Supplementary material for: DNA barcoding of Malaysian commercial snapper reveals an unrecognized species of the yellow-lined Lutjanus (Pisces:Lutjanidae)
Source: PLoS One. 2018 Sep 5;13(9):e0202945. doi: 10.1371/journal.pone.0202945 (PMC6124743; doi:10.1371/journal.pone.0202945)

**S2 APPENDIX**

**Species composition of specimens obtained.**

S2 Table: Species composition according to specimen numbers obtained and life stages (adult or juvenile).


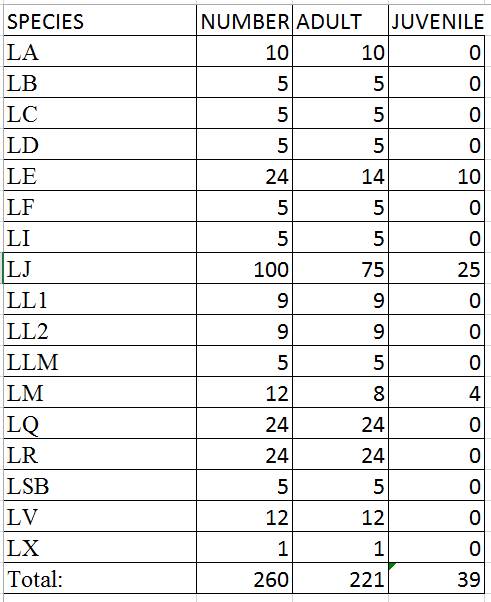


| LA-*L.argentimaculatus* | LL2-*L.lutjanus 2* |
| --- | --- |
| LB-*L.bohar* | LLM-*L.lemniscatus* |
| LC-*L.carponotatus* | LM-*L.malabaricus* |
| LD-*L.decussatus* | LQ-*L.quinquelineatus* |
| LE-*L.erythropterus* | LR-*L.russelli* |
| LF-*L.fulviflamma* | LSB-*L.sebae* |
| LI-*L.indicus* | LV-*L.vitta* |
| LJ-*L.johnii* | LX-*L.xanthopinnis* |
| LL1-*L.lutjanus 1* |  |

S2 Figure (a): Pie chart showing percentage of fishes obtained in overall sampling according to species.


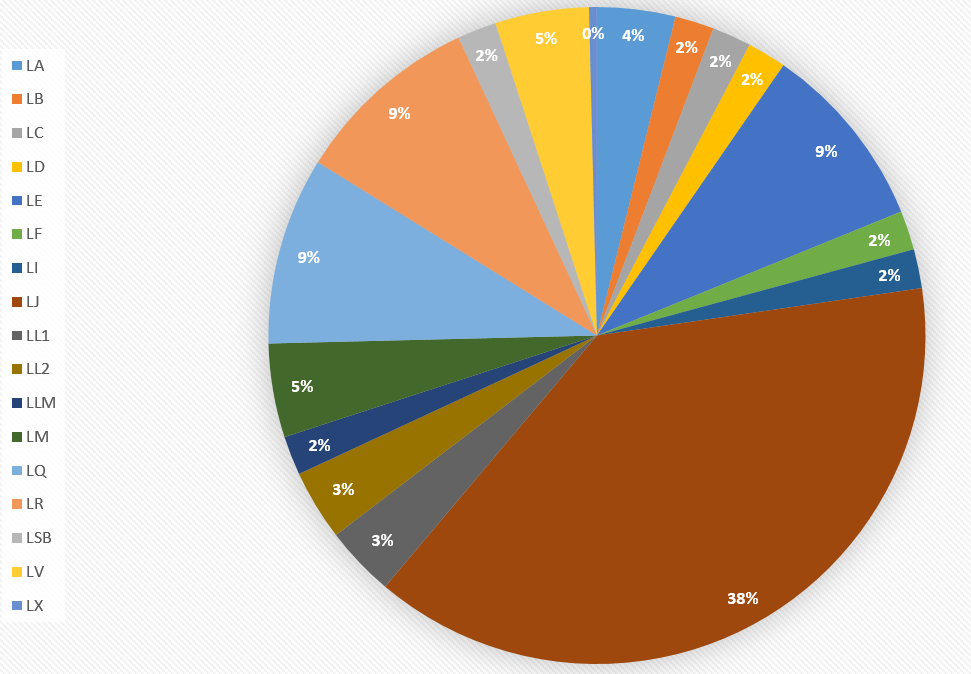


S2 Figure (b): Bar graph showing number of specimen collected according to species and life stages (adult or juveniles).


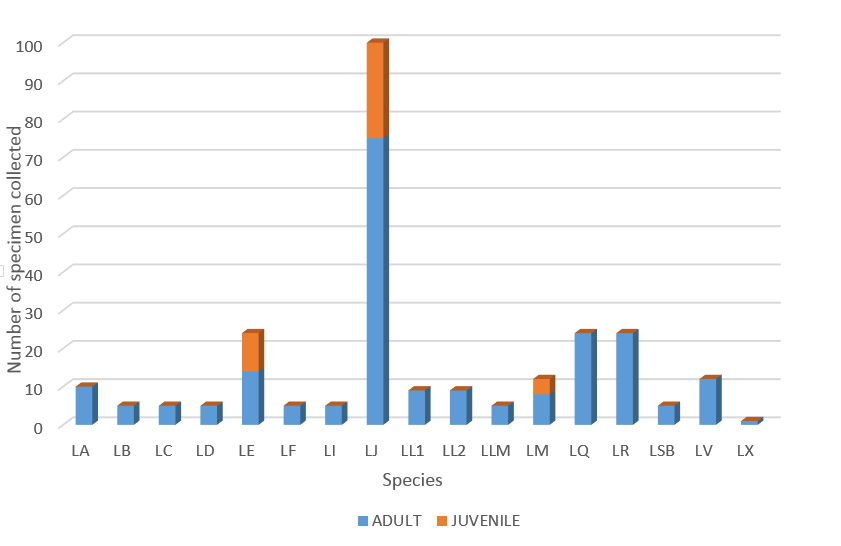

Supplement: S2 Appendix — (DOC) [file pone.0202945.s002.doc]
